# Unraveling the genetic diversity and phylogeny of *Leishmania* RNA virus 1 strains of infected *Leishmania* isolates circulating in French Guiana

**DOI:** 10.1371/journal.pntd.0005764

**Published:** 2017-07-17

**Authors:** Sourakhata Tirera, Marine Ginouves, Damien Donato, Ignacio S. Caballero, Christiane Bouchier, Anne Lavergne, Eliane Bourreau, Emilie Mosnier, Vincent Vantilcke, Pierre Couppié, Ghislaine Prevot, Vincent Lacoste

**Affiliations:** 1 Laboratoire des Interactions Virus-Hôtes, Institut Pasteur de la Guyane, Cayenne, French Guiana; 2 Ecosystèmes Amazoniens et Pathologie Tropicale, EA 3593, Medicine Department, University of French Guiana, Cayenne, French Guiana; 3 National Reference Center for Leishmania, associated laboratory, Laboratory of Parasitology and Mycology, Centre Hospitalier Andrée Rosemon, Cayenne, French Guiana; 4 Bioinformatics Graduate Program, Boston University, Boston, MA, United States of America; 5 Plate-forme Génomique, Pôle Biomics, Institut Pasteur, Paris, France; 6 Laboratoire d’Immunologie des Leishmanioses, Institut Pasteur de la Guyane, Cayenne, French Guiana; 7 Unité des Maladies Infectieuses et Tropicales, Centre Hospitalier Andrée Rosemon, Cayenne, French Guiana; 8 Centres Délocalisés de Préventions et de Soins, Centre Hospitalier Andrée Rosemon, Cayenne, French Guiana; 9 Department of Medicine, Centre Hospitalier de l'Ouest Guyanais, Saint Laurent du Maroni, French Guiana; 10 Dermatology Unit, Centre Hospitalier Andrée Rosemon, Cayenne, French Guiana; 11 Department of Virology, Institut Pasteur, Paris, France; IRD, FRANCE

## Abstract

**Introduction:**

*Leishmania* RNA virus type 1 (LRV1) is an endosymbiont of some *Leishmania* (*Vianna*) species in South America. Presence of LRV1 in parasites exacerbates disease severity in animal models and humans, related to a disproportioned innate immune response, and is correlated with drug treatment failures in humans. Although the virus was identified decades ago, its genomic diversity has been overlooked until now.

**Methodology/Principles findings:**

We subjected LRV1 strains from 19 *L*. *(V*.*) guyanensis* and one *L*. *(V*.*) braziliensis* isolates obtained from cutaneous leishmaniasis samples identified throughout French Guiana with next-generation sequencing and *de novo* sequence assembly. We generated and analyzed 24 unique LRV1 sequences over their full-length coding regions. Multiple alignment of these new sequences revealed variability (0.5%–23.5%) across the entire sequence except for highly conserved motifs within the 5’ untranslated region. Phylogenetic analyses showed that viral genomes of *L*. *(V*.*) guyanensis* grouped into five distinct clusters. They further showed a species-dependent clustering between viral genomes of *L*. *(V*.*) guyanensis* and *L*. *(V*.*) braziliensis*, confirming a long-term co-evolutionary history. Noteworthy, we identified cases of multiple LRV1 infections in three of the 20 *Leishmania* isolates.

**Conclusions/Significance:**

Here, we present the first-ever estimate of LRV1 genomic diversity that exists in *Leishmania (V*.*) guyanensis* parasites. Genetic characterization and phylogenetic analyses of these viruses has shed light on their evolutionary relationships. To our knowledge, this study is also the first to report cases of multiple LRV1 infections in some parasites. Finally, this work has made it possible to develop molecular tools for adequate identification and genotyping of LRV1 strains for diagnostic purposes. Given the suspected worsening role of LRV1 infection in the pathogenesis of human leishmaniasis, these data have a major impact from a clinical viewpoint and for the management of *Leishmania*-infected patients.

## Author summary

Leishmaniasis is a well-known parasitosis due to an infection by the protozoan *Leishmania* parasites firmly established in South America. In French Guiana, where leishmaniasis is a public health problem, having an annual incidence of 5.6 cases/10,000 inhabitants, 80% of *Leishmania spp*. parasites are infected by an endosymbiotic virus, *Leishmania* RNA virus 1 (LRV1). The purpose of this study was to gain insights on the genetic variability and evolution of LRV1 at the genomic level. We subjected 20 *Leishmania* isolates obtained from cutaneous lesions with next generation sequencing and *de novo* sequence assembly. This allowed generating 24 LRV1 full-length coding sequences presenting among themselves a significant genetic diversity. The inferred phylogenetic relationships enabled to identify distinct well-supported genotypes and support the hypothesis of co-evolution between LRV1 strains and their parasite hosts. In addition, we identified multiple LRV1 infections in three parasite isolates. Based on these data, future characterization of new strains from other geographic areas and other parasite species should extend knowledge of LRV1 diversification processes. Finally, these results allowed us to identify genomic regions where best to allocate resources for subsequent analyses of LRV1 viral diversity and genotyping that could serve for accurate routine molecular diagnostic applications.

## Introduction

Protozoan parasites of the genus *Leishmania* are unicellular eukaryotes with a complex digenetic cycle. In the vertebrate host, they are obligatory intracellular parasites. They cause a broad spectrum of diseases, collectively known as leishmaniases, that occur predominantly in tropical and subtropical regions [1]. Given their frequency and the severity of certain clinical forms, these diseases represent a major public health problem in endemic countries. Depending on their behavior in the vector’s gut, parasites of the *Leishmania* genus are divided into two subgenera: *Leishmania* and *Viannia*. The *Leishmania* subgenus *Viannia*, endemic in Latin America, is the etiological agent of cutaneous (CL) and mucocutaneous leishmaniasis (MCL) [2]. Within this subgenus, *L*. *(V*.*) guyanensis* and *L*. *(V*.*) braziliensis* are hosts of a double-stranded RNA virus called *Leishmania* RNA Virus (LRV).

*Leishmania* RNA viruses are small, nonenveloped double-stranded RNA viruses [3]. LRV (genus *Leishmaniavirus*) are members of the *Totiviridae* family, along with several other groups of protozoal or fungal viruses, including *Giardia lamblia* virus (GLV), *Trichomonas vaginalis* viruses and *Eimeria* spp. viruses [3–9]. They have a nonsegmented genome approximately 5 kb (4.9–5.3 kb) in length with two long, partially overlapping, open reading frames on the positive strand encoding the capsid/coat protein (CP; *orf2*) and the RNA-dependent RNA polymerase (RdRp; *orf3*) and a small 5’-proximal potential *orf1* [10]. LRV *orf1* presents similarities with *orf1* of ScV-L-A virus, a totivirus of the yeast *Saccharomyces cerevisiae* that encodes a toxin, but its function remains unsolved. LRV genomic RNA is flanked at its extremities by 5’ and 3’ untranslated regions (UTR). It has been shown that the 5’UTR promotes internal initiation of translation with the presence of an internal ribosomal entry site (IRES) [11]. The 5’UTR also possesses five predicted stem-loop structures (I to V) and a consensus cleavage sequence [12–14]. Stem-loop IV and the consensus cleavage site represent the minimal essential components for the viral capsid-dependent RNA cleavage that might play a regulatory role for maintaining persistent infection of host cells [13, 15].

The presence of virus-like particles in *Leishmania* parasites was first reported in the early 1970s [16]. Since then, two types of LRV have been identified: LRV1 carried by some New World *Leishmania* species and LRV2 by Old World species [3, 8, 17]. To date, LRV1 was detected in *Leishmania* parasites from Colombia, Brazil, Peru, Bolivia, Suriname and French Guiana, all originating in the Amazon basin [18–22]. LRV2 was first isolated from *L*. *hertigi*, a non-human parasite species [16]. It was then found in a single isolate of *L*. *major* and, more recently, in strains of *L*. *aethiopica* isolated from biopsies of cutaneous leishmaniasis patients in the Ethiopian highlands as well as in two different *Leishmania* isolates from Iran belonging to the *L*. *infantum* and *L*. *major* species [17, 23, 24]. At the time of their discovery, arbitrary identifiers were given to type 1 *Leishmania* RNA viruses showing a certain degree of sequence conservation on the basis of hybridization analysis, namely LRV1-1 to LRV1-12 [20]. Two additional types, LRV1-13 and LRV1-14, were then described based on sequence comparison of fragments of two genomic regions, i.e., the 5’UTR and *orf3* regions [25]. Several subtypes were isolated from *L*. *(V*.*) guyanensis*, LRV1-(1, 4, 5, 6, 7, 8 and 9), others from *L*. *(V*.*) braziliensis*, LRV1-(2, 3, 13 and 14) and untyped *Leishmania* spp., LRV1-(10, 11, 12). In addition to *L*. *(V*.*) guyanensis* and *L*. *(V*.*) braziliensis*, Cantanhêde et al. recently reported the identification of LRV1 in *L*. *(V*.*) lainsoni* and *L*. *(Leishmania) amazonensis* parasites [26]. So far, no LRV1 has been detected in other *L*. *(V*.*)* species and none in *L*. *(L*.*)* species [18, 21]. These results, proving the wide distribution of LRV1 in the different New World parasite species, combined with the lack of an infectious phase for these viruses, suggested that LRV1 arose prior to the divergence of New World parasites and further supported the theory that LRVs are ancient viruses [25]. Nevertheless, most of these viruses have only been characterized on short stretches of sequences a few hundred base-pairs long and, depending on the studies, different regions of the genome have been amplified. Complete cDNA sequences are only available for two New World virus isolates (LRV1-1 and LRV1-4), one LRV2-1 isolated from *L*. *major* and three more LRV2-1 strains from *L*. *aethiopica* [10, 12, 23, 27]. The LRV1-1 and LRV1-4 entire genomic sequences share 77% overall nucleotide identity.

Little is known on the biology of LRV, which persistently infects *Leishmania* parasites and is unable to produce extracellular infectious particles. Nevertheless, LRV1 infection of parasites seems to affect their virulence. Indeed, Ives et al. showed that metastasizing parasites have a high LRV1 burden that is recognized by host Toll-like receptor 3 (TLR3) to induce proinflammatory cytokines and chemokines in murine models [28]. They also showed that LRV1 in the metastasizing parasites subverted the host immune response to *Leishmania*, promoting parasite persistence and affecting treatment efficiency. We and others recently showed that LRV1 status in *L*. *(V*.*) guyanensis*- and *L*. *(V*.*) braziliensis*-infected patients was significantly predictive of first-line treatment failure and symptomatic relapse, and might stand to guide therapeutic choices in acute cutaneous leishmaniasis (ACL) [29, 30]. However, other studies reported contradictory results with respect to the association between the severity of leishmaniasis (cutaneous *vs*. mucocutaneous) and the presence of LRV1 [19, 22, 26, 31, 32].

In French Guiana, where CL is a public health problem with an average of 127 diagnosed cases per year between 2006 and 2013, oscillating between 96 and 160 cases per year, five coexisting *Leishmania* parasite species are known to infect humans: *L*. *(V*.*) guyanensis*, *L*. *(V*.*) braziliensis*, *L*. *(L*.*) amazonensis*, *L*. *(V*.*) lainsoni* and *L*. *(V*.*) naiffi* [33, 34]. Among them, *L*. *(V*.*) guyanensis* and *L*. *(V*.*) braziliensis* are the two predominant species, accounting for more than 85% and about 10% of CL cases, respectively [33]. The three other species are only occasionally diagnosed [33, 35]. For *L*. *(V*.*) guyanensis* and *L*. *(V*.*) braziliensis*, patients present a broad spectrum of clinical manifestations (nodular, ulcerative, disseminated and mucous forms). In addition, symptomatic relapses and treatment failures are frequently seen in *L*. *(V*.*) guyanensis* infections during which patients variously respond to first-line anti-leishmanial treatments and are more prone to developing chronic CL. Factors underlying disease evolution are yet to be fully understood. We recently reported that 74% of *Leishmania* spp. isolates collected between 2011 and 2014 were LRV1-positive [21]. LRV1 was detected in 80% (90/112) of *L*. *(V*.*) guyanensis* isolates, 55% (6/11) of *L*. *(V*.*) braziliensis* and in none of the (0/6) *L*. *(L*.*) amazonensis* isolates. Considering the paucity of sequence data available for LRV1, because of the many questions that arise about its origin and distribution, we were interested in gaining insight into the genetic diversity, at the genomic level, and the evolution of this virus in the circulating isolates of *Leishmania* spp. in French Guiana. To this end, using Illumina deep-sequencing technology and *de novo* sequence assembly, we generated and analyzed LRV1 sequences derived from *Leishmania* parasite cultures obtained from skin lesions of 20 patients suffering of ACL, i.e., with lesions present for less than 6 months. Polymorphism and phylogenetic analyses were then performed to measure LRV1 genome-wide diversity. Here we report the full-length coding sequences of 24 LRV1 strains and use them to make the first-ever estimate of LRV1 viral diversity that exist in *Leishmania (V*.*) guyanensis*. These results represent the largest number of LRV1 full-length coding sequences published to date and the first time that in some parasite strains multiple viral infections have been identified.

## Materials and methods

### Ethics statement

The *Leishmania* isolates used in this study were obtained from a previously published study [21]. Briefly, these isolates had been successfully cultured from biopsies collected between 2011 and 2014 from 20 adult patients (16 males and four females; 19–66 years old) diagnosed with ACL and enrolled in the study before treatment with pentamidine isethionate (Pentacarinat; Rhone Poulenc) (**Table 1**). All patients, in consultation for a suspicion of leishmaniasis at the dermatology unit of the Cayenne hospital (Centre Hospitalier Andrée Rosemon, CHAR), Cayenne, French Guiana, were informed during consultation by the clinician that case records and biological data might be further used in research and that they had the right to refuse. Biological samples were taken for diagnostic purposes and oral informed consent was documented in the case records by the clinician during consultation. Patient data was anonymized at the Laboratory of Parasitology and Mycology of the Cayenne hospital, which carried out diagnostic analyzes and initial parasite culture. The project did not raise any concerns and was approved by the Ethical Committee at Cayenne Hospital. Ethical approval was granted based on the human experimentation guidelines of the “Comité consultatif sur le traitement de l'information en matière de recherche” (CCTIRS: 2012–42). The monocentric audit of retrospective anonymized case record data was permitted by the “Commission nationale de l’informatique et des libertés” (CNIL: DR.2014-091) as well as the regulations of Cayenne Hospital (http://www.ch-cayenne.net/Droits-et-Devoirs.html).

**Table 1:** Epidemiological features of the 20 patients with ACL, *Leishmania* species identification and characteristics of the 24 LRV1 strains sequenced.

	Patient				Parasite	LRV1								
Strain ID^a^	Gender	YOB^b^	CD^c^	Age	Species ID^d^	Clade^e^	Genome^f^	5'UTR^f^	ORF2/CP^f^	ORF3/RdRp^f^	3'UTR^f^	G+C%	International code	Accession no.
2008	F	1993	2012	19	*L*. *guyanensis*	A	5268	389	2229	2637	13	0.454	LRV1-Lg-2008	KY750612
2015	M	1972	2012	40	*L*. *guyanensis*	A	5268	389	2229	2637	13	0.455	LRV1-Lg-2015	KY750613
WF69_G1_	M	1988	2012	24	*L*. *guyanensis*	A	5268	389	2229	2637	13	0.455	LRV1-Lg-WF69_G1_	KY750624
WF69_G2_					* *	A	5268	389	2229	2637	13	0.464	LRV1-Lg-WF69_G2_	KY750625
XK73	M	1990	2013	23	*L*. *guyanensis*	A	5268	389	2229	2637	13	0.459	LRV1-Lg-XK73	KY750627
LF98	F	1974	2013	39	*L*. *guyanensis*	A	5268	389	2229	2637	13	0.454	LRV1-Lg-LF98	KY750617
YR07	M	1984	2014	30	*L*. *guyanensis*	A	5268	389	2229	2637	13	0.455	LRV1-Lg-YR07	KY750629
2028_G2_					* *	A	5268	389	2229	2637	13	0.456	LRV1-Lg-2028_G2_	KY750615
2028_G3_					* *	A	5268	389	2229	2637	13	0.464	LRV1-Lg-2028_G3_	KY750616
2028_G1_	F	1971	2012	41	*L*. *guyanensis*	B	5268	389	2229	2637	13	0.458	LRV1-Lg-2028_G1_	KY750614
LL28	M	1960	2012	52	*L*. *guyanensis*	B	5268	389	2229	2637	13	0.457	LRV1-Lg-LV11	KY750618
2014	M	1983	2013	30	*L*. *guyanensis*	B	5267	388	2229	2637	13	0.46	LRV1-Lg-2014	KY750611
YE48	M	1986	2013	27	*L*. *guyanensis*	B	5268	389	2229	2637	13	0.459	LRV1-Lg-YE48	KY750628
PD46	F	1948	2014	66	*L*. *guyanensis*	B	5268	389	2229	2637	13	0.455	LRV1-Lg-PD46	KY750621
VW21	M	1987	2013	26	*L*. *guyanensis*	B	5268	389	2229	2637	13	0.459	LRV1-Lg-VW21	KY750623
MJ25	M	1976	2012	36	*L*. *guyanensis*	B	5268	389	2229	2637	13	0.462	LRV1-Lg-MJ25	KY750620
MC71	M	1993	2012	19	*L*. *guyanensis*	B	5268	389	2229	2637	13	0.462	LRV1-Lg-MC71	KY750619
YZ58	M	1959	2012	53	*L*. *guyanensis*	B	5268	389	2229	2637	13	0.457	LRV1-Lg-YZ58	KY750630
VL91	M	1977	2012	35	*L*. *guyanensis*	B	5268	389	2229	2637	13	0.454	LRV1-Lg-VL91	KY750622
XJ93_G1_	M	1978	2013	35	*L*. *guyanensis*	B	5268	389	2229	2637	13	0.458	LRV1-Lg-XJ93_G1_	KY750626
XJ93_G2_					* *	C	5269	390	2229	2637	13	0.458	LRV1-Lg-XJ93_G2_	KY750609
LF94	M	1955	2013	58	*L*. *guyanensis*	D	5266	387	2229	2637	13	0.466	LRV1-Lg-LF94	KY750608
2001	M	1978	2011	33	*L*. *guyanensis*	E	5270	391	2229	2637	13	0.447	LRV1-Lg-2001	KY750607
YA70	M	1983	2013	30	*L*. *braziliensis*	F	5262	389	2229	2631	13	0.459	LRV1-Lb-YA70	KY750610

^a^ ID, identification;

^b^ YOB, year of birth;

^c^ CD, collection date.

^d^ as formely determined by PCR-RFLP RPOF2/R2

^e^ based on sequence comparison and phylogenetic analysis of the complete coding sequences of CP and RdRp.

^f^ sizes in nucleotides

### LRV1 status and parasite species confirmation

All 20 isolates of *Leishmania* spp. had formerly been tested for their LRV1 status by RT-PCR and *Leishmania* spp. were identified using PCR-RFLP, as previously described (**Table 1**) [21, 36].

### Parasite cultures

Parasites were cultured in Schneider’s medium (Sigma-Aldrich) complemented with 10% heat-inactivated fetal calf serum (FCS) (Gibco), 0.6 mg/L Biopterin (Sigma-Aldrich) and 5 mg/L folate hemin (Sigma-Aldrich) without antibiotics. Dry pellets were constituted by centrifugation 5 min at 2500 rpm and removal of supernatant. The pellets were stored at −80°C until RNA extraction.

### Biological cloning

For parasite cloning, 0.5 mL of culture medium containing 2–4.10^7^ parasites was spread onto freshly prepared Schneider plates (Schneider’s medium, 10% FCS, Biopterin 0.6 mg/L and folate hemin 5 mg/L, 2% low-melting-point agarose). After 7 days, the colonies were harvested and cultured separately in tubes containing complemented Schneider’s medium for an additional 5–7 days. Separate cultures were then processed for RNA isolation and conventional RT-PCR using primer pairs specific to each virus (see below).

### RNA extraction and sequence-independent amplification

Total RNA was extracted from dry pellets of parasites (10^8^ parasites) using the RNeasy mini kit (QIAGEN) as recommended by the manufacturer, treated with Turbo DNase (Life Technologies) for 1 h at 37°C and then measured on a Biophotometer (Eppendorf). Then the generation of double-stranded cDNA and SPIA amplification was performed from 50 ng of total RNA using the Ovation RNA-Seq system V2 (NuGEN Technologies, Inc.) as specified by the manufacturer’s protocol.

### Generation of libraries and high-throughput sequencing

The libraries were generated using the NextFlex PCR-free DNA Seq Kit (Bioo Scientific, Austin, TX, USA) with (15 strains, with a two-letter and two-number code) or without (five strains, with a 20XX code) a 10-cycle PCR enrichment before quantification and validation. They were then sequenced on an Illumina MiSeq instrument in 250-base paired-end reads (Illumina, San Diego, CA, USA). Sequence files were generated using Illumina Analysis Pipeline version 1.8 (CASAVA).

### Bioinformatics analysis

Raw sequences were filtered and trimmed using fastq-mcf with the following parameters: duplicates were removed when 50 bases were identical between reads (-D parameter) while minimum read length was set to 50 [37]. Adapter sequences were removed using -t (% of occurrence) set to zero. The other parameters were left to their default settings. Then Deconseq was used to eliminate phiX174 phage sequences [38]. Fastq clean files were assembled using SPAdes (version 3.0.0) with the following range of k-mers: 21, 55, 77, 99 and 127 [39]. We used blastn for sequence comparison between assembled contigs and reference LRV1 genomes (LRV1-1 and LRV1-4, accession numbers M92355 and U01899, respectively) [6]. The LRV1-positive contigs were then extracted and used as references for mapping using the bwa mem algorithm (version 0.7.10-r789) with a kmer set to 55 and a minimum alignment score of 40, using a minimum fastq quality of 30 [40]. Some assembly abnormalities were manually corrected. The contigs and known LRV genomes were then multiple-aligned with MAFFT alignment software (version 7.037b) [41].

### Conventional RT-PCR and Sanger sequencing

Conventional methods were used to extend sequences when needed. Three sets of degenerate consensus primers were designed according to the NGS sequences obtained. Primers were degenerated so as to amplify products from all samples regardless of the sequence. Full information on the primers is available in **Table 2**. These sets of primers were used in RT-PCR reactions carried out using the Transcriptor one-step RT-PCR kit (Roche), as recommended by the manufacturer, using 500 ng of input total RNA. RT-PCR products at the expected size were sent to Beckman Coulter Genomics (http://www.beckmangenomics.com/) for direct sequencing. Furthermore, to specifically amplify the different viruses in case of multiple-infected *Leishmania* isolates, one set of nondegenerate specific primers per virus was designed and used for RT-PCR (**Table 2**).

**Table 2:** Sequences of oligonucleotide primers used for consensus and specific RT-PCRs.

	Oligonucleotide	Orientation^*a*^	5’ -> 3’ sequence^*b*^	Position^*c*^	Ta^*d*^	Elongation	Size^*e*^
*Degenerate primers*							
	LRV1s	+	ATTCGCTAGCTGTYBGGATGGTAGYGTTAC	30–59	60°C	1 min	779
	LRV2as	-	CATAGCCAAAACGTTCACAWARTGTYGRGTGT	778–809			
	LRV3s	+	ATGCATGTHGGTGATGACATHYTRATGTC	4258–4286	50°C	1 min	922
	LRV4as	-	TGAGCCATTGARGTYGCTTCRTTRTAYGGA	5151–5180			
	5LRVs1	+	ATCATGGCCCAGGCYAGCTG	4840–4859	50°C	30 sec	438/432
	LRV11as	-	ATAGTTTATGRCACTYTCTGCCATATTCC	5249–5277			
	LRV14as	-	TTATGGCACTYTTGCCATATTCC	5249–5272			
*Specific primers*							
	XJ93_G1_F	+	TGTCCCGATTGCTGGTTACT	1307–1326	55°C	1 min	656
	XJ93_G1_R	-	GGACGCAACCTGAAATCTACGTTAG	1939–1963			
	XJ93_G2_F	+	ACATGCCTTGGACCAAAACC	4079–4098	55°C	30 sec	251
	XJ93_G2_R	-	TGGGTCAGGTTAGCTATTAGGT	4309–4330			
	WF69_G1_F	+	CTGACTTAGGTGGGTATGCAA	2960–2980	55°C	1 min	606
	WF69_G1_R	-	TAGACTTGACGCACTGCCA	3548–3566			
	WF69_G2_F	+	CTGACTTGGGAGGGTACGTGA	2960–2980	55°C	1 min	639
	WF69_G2_R	-	GGGTACGAAACAGACCTTTTAATCT	3575–3599			

^*a*^ +, sense; -, antisense.

^*b*^ Positions of degeneracy follow the IUPAC-IUB (International Union of Pure and Applied Chemistry-International Union of Biochemistry) codes.

^*c*^ Position of primers are indicated relative to the LRV1-1 sequence, GenBank accession number M92355.

^*d*^ Ta, annealing temperature.

^*e*^ Size of PCR products in base pairs.

### Phylogenetic analyses

Contigs from HTS as well as raw sequences downloaded from the Beckman Coulter Genomics website were analyzed and edited in MEGA 5.05 [42]. Multiple sequence alignments were constructed using ClustalW with all published complete and partial LRV1 sequences. Alignments were checked manually. Sequences were translated into amino acids and both nucleotide and amino acid sequences were checked for irregularities. For phylogenetic trees inferred from the aligned nucleotide sequences, the MrModeltest2.3 program was used to determine the optimal model of nucleotide evolution [43]. The GTR model, with a gamma distribution shape parameter (G) and invariable sites (I), was identified and used for the Bayesian approach, which was performed with Mr. Bayes 3.2.2 to infer phylogenetic relationships [44]. Markov Chain Monte Carlo (MCMC) simulations were run for 10,000,000 generations, with four simultaneous chains, using a sample frequency of 100 and a burn-in of 25,000. Majority rule consensus trees were obtained from the output. Validation of the inference was assessed based on the standard deviation of split frequencies, which was less than the expected threshold value of 0.01. For phylogenetic trees inferred from the aligned amino acid sequences, the ProtTest3 program was used to determine the optimal model of amino acid evolution. The JTT model, with gamma (G) distribution, was identified and used for the Bayesian approach [45], which was performed with Mr. Bayes 3.2.2 [44]. MCMC simulations were run for 10,000,000 generations, with four simultaneous chains, using a sample frequency of 100 and a burn-in of 25,000. Majority rule consensus trees were obtained from the output. Validation of the inference was assessed based on the standard deviation of split frequencies, which was less than the expected threshold value of 0.01.

### Naming assignation

LRV1 strains were named according to a recent proposal to the International Committee on Taxonomy of Viruses, i.e., “LRV1” followed by two letters corresponding to the parasite species to which the strain designation was then assigned [46]. For example: LRV1-Lg-LF94 for LRV1 from *L*. *(V*.*) guyanensis* strain LF94 (**Table 1**).

### Accession numbers

Sequences reported in this paper were deposited in the GenBank nucleotide database under accession numbers **KY750607** to **KY750630**. The raw sequencing data are available in the Sequence Read Archive under accession number **PRJNA371487** (www.ncbi.nlm.nih.gov/bioproject/371487).

## Results

### Patients

In the context of epidemiological screening, we tested 129 isolates of *Leishmania* sp. Collected in French Guiana between 2011 and 2014 for the presence of LRV1 [21]. Here, we analyzed a subset of previously identified LRV1-positive isolates obtained from patients diagnosed with ACL. A total of 20 parasite cultures, obtained from human biopsies isolated from 16 men and four women, aged 19–66 years with a median age of 35 years, were selected for high-throughput sequencing. The data (gender and age) collected for each patient are listed in **Table 1**. In all, we sequenced 19 LRV1-positive *L*. *(V*.*) guyanensis* isolates and one LRV1-positive *L*. *(V*.*) braziliensis* isolate.

### Global analysis of high-throughput sequencing data

Using Illumina MiSeq sequencing technology, 24 full-length or almost full-length genomic sequences were *de novo* assembled into individual contigs with a mean 49.8 depth of coverage. Sequences were 4.8–5.3 kb long. Alignment of the full-length genome sequences obtained with those available in the database made it possible to design three sets of consensus-degenerate primers (**Table 2**). These primer pairs were used for conventional RT-PCR and Sanger sequencing to extend the sequences to the 5’ and 3’ ends that were missing for some strains. High-throughput sequencing combined with conventional RT-PCR applied to the 20 LRV1-positive isolates generated almost complete genomic sequences (from nucleotide 60 to 5248 of LRV1-1, accession number NC_002063) from each viral strain. This covers the full-length coding sequence of the virus. In addition, Sanger sequencing of the RT-PCR products also allowed verifying the correctness of the sequences obtained. A total of 24 different viral sequences were obtained from the 20 LRV1-positive parasite isolates tested. All sequences were 5.2 kb long with an average G+C content ranging from 44.7% to 46.6%. Detailed genome statistics are outlined in **Table 1**. Unexpectedly, co-infections by two or three LRV1 viruses were identified in three parasite isolates, 2028, WF69 and XJ93. LRV1 full-length coding sequences obtained from the same isolate were named 2028_G1_, 2028_G2_ and 2028_G3_, WF69_G1_ and WF69_G2_, XJ93_G1_ and XJ93_G2_.

### Sequence features

The 24 sequences obtained had the same structural organization as partially overlapping open reading frames surrounded by untranslated regions similar to the sequence already described [10, 12]. In addition, they showed an identical genome size. All but one sequence (strain LF94) showed a double-nucleotide insertion in the 5’UTR region at positions 201–202 relative to LRV1-1 (numbers given here and below refer to the nucleotide positions in the published sequence of LRV1-1, acc. # NC_002063). At this exception of this shared insertion, only a few other nucleotide insertions and deletions were seen in the 5’UTR region of four viral sequences relative to LRV1-1. Thus, strain 2014 exhibited one nucleotide deletion at position 363, strain 2001 possessed two nucleotide insertions at positions 142 and 198 (these two insertions were also present in LRV1-2, LRV1-8 and LRV1-9 5’UTR sequences), strain XJ93_G2_ showed a double-nucleotide insertion at position 198–199 and one nucleotide deletion at position 446, while strain YA70 presented one nucleotide insertion at position 198 and one deletion at position 393. In addition to these indels in the 5’UTR, of the 24 sequences, only the coding sequence of the *RNA-dependent RNA polymerase* gene of the YA70 strain presented one codon insertion at positions 73–75 (D25), one codon deletion at positions 169–171 and a double codon deletion at positions 493–498. These codon indels did not alter the reading frame. YA70 shared the first amino acid insertion at position 25 (aspartic acid or lysine, respectively) and the first codon deletion with strain LRV1-Lb2169 (international code MHOM/BO/2011/2169, acc. # KC862308). These two strains were identified from *L*. *(V*.*) braziliensis* parasites. The YA70 strain also possessed a double stop codon at the end of the *coat* and *RNA-dependent RNA polymerase* genes. Three other strains also possessed a double stop codon at the end of the *RNA-dependent RNA polymerase* gene: LRV1-1, LF94 and 2001. Except for YA70, no deletions or insertions were detected in the coding sequences of the other strains. According to these results, *orf2* was 2229 bp in length and *orf3* was 2631–2637 bp, encoding a capsid protein of 742 aa and a RNA-dependent RNA polymerase of 876–878 aa, respectively.

### Sequence identities

The almost complete genome sequences obtained for each strain as well as the nucleotide and amino acid sequences of the capsid and RNA-dependent RNA polymerase were compared to one another and to those published in the databases. All sequences were unique. On the basis of the common ≈ 5260 bp sequences obtained for each strain, pairwise comparisons showed that nucleotide sequence identities ranged from 76.5% (2001 *vs*. LRV1-4) to 99.5% (PD46 *vs*. VL91). CP coding sequences exhibited among themselves from 76.3% (Lg1398 *vs*. LRV1-Lb2169) to 99.7% (2008 *vs*. LF98) nucleotide identity and from 89.5% (LRV1-4 *vs*. LRV1-Lb2169) to 100% (PD46 *vs*. VL91 and 2008 *vs*. LF98 and 2028_G1_ *vs*. LL28/LV11) amino acid identity. For RdRp coding sequences, the nucleotide identities ranged from 72.4% (2001 *vs*. LRV1-Lb2169) to 99.4% (PD46 *vs*. VL91) and the amino acid identities ranged from 79.9% (2001 *vs*. LRV1-4 *vs*. LRV1-Lb2169) to 99.5% (2008 *vs*. LF98). Based on the genetic distance (**Table 3**) and phylogenetic relationship results (see below), six clades were clearly identifiable. We tentatively called them clades A–F. With the exception of clade A sequences for which the nucleotide divergence ranged from 0.3 to 10.4%, the intra-clade variability of all clades was below 10% at both the nucleotide and amino acid levels. Moreover, except for the A-B inter-clade variability, which ranged from 95.5 to 98.2% on CP and from 92.3 to 94.9% on RdRp on amino acid sequences, the inter-clade variability was over 10% at the nucleotide and amino acid levels. It is noteworthy that the two LRV1 sequences obtained from isolate WF69, WF69_G1_ and WF69_G2_, had a 91.8% nucleotide identity. Strains 2028_G2_ *vs*. 2028_G3_ gave the same percentage of nucleotide identity (91.8%), while 2028_G1_ *vs*. 2028_G2_ and 2028_G1_ *vs*. 2028_G3_ showed 86.9 and 87.4% nucleotide identity, respectively. Finally, strains XJ93_G1_ *vs*. XJ93_G2_ exhibited 83.4% nucleotide identity. Nucleotide substitutions occurred throughout the sequences.

**Table 3:** Nucleotide and amino acid identities between the novel LRV1 sequences and those available in the databases, as reported by clades (A-F).

**A-**													
	% Identity with												
		A	B	C	D	E	F										
	A	90.9–98.8											
	B	86.4–87.6	92.7–99.5										
	C	82.9–83.9	83.4–83.6	-									
	D	77.5–78.6	77.9–78.8	78–78.5	96.8								
	E	76.5–77.2	76.7–77.4	77.7	77.6–77.8	-							
	F	77.2–77.7	77.3–78.1	77.7	77.7–77.9	77.3	-						
**B-**													
		% Identity with											
		A		B		C		D		E		F	
		nct	aa	nct	aa	nct	aa	nct	aa	nct	aa	nct	aa
	A	89.6–99.7	95.7–100										
	B	85.6–87.4	95.5–98.2	91.8–99.6	98.5–100								
	C	82.3–83.2	94.5–96.5	82.4–83.3	95.8–96.5	-	-					
	D	78.4–79.4	90.8–92.7	78.2–79.3	91.9–92.4	78.3–78.6	92.8–93.1	96.7	99.5			
	E	77.8–79.5	89.9–91.9	77.7–79.5	91.8–92.4	78.8–79.1	91.6–92	78.6–79.3	91.6–92	98.2	99.3		
	F	77.3–78.5	89.5–92.4	78–79.5	90.8–92.4	77.9–78.8	91.5–92.4	77.8–78.7	91.8–92.4	76.3–78.5	90.7–91.4	80.7	95.5
**C-**													
		% Identity with											
		A		B		C		D		E		F	
		nct	aa	nct	aa	nct	aa	nct	aa	nct	aa	nct	aa
	A	90.4–99	94.9–99.5										
	B	85.4–87.1	92.3–94.9	92.4–99.4	95.8–99.4								
	C	81.5–83.1	88.1–89.5	81.9–82.7	88.9–89.6	-	-						
	D	74.9–76.5	82.2–83.9	75.2–76.7	82.7–84	75.6–76.3	83.8–84.2	96.5	97.7				
	E	72.9–74.3	79.9–81.5	73.3–73.9	80.7–81.1	74.6	81.3	74.8–75	81.7–81.8	-	-		
	F	73.7–75.9	81.9–83.9	74.7–75.4	81.8–83.7	75–75.1	82.6–82.9	74–75.4	80.8–81.8	72.4–74.7	79.9–80.7	78.8	86.3

A- Numbers refer to values obtained by comparing the common 5176 base-pair sequences of the obtained LRV1 sequences with the few other LRV1 sequences available.

B- Numbers refer to values obtained by comparing the complete coding sequences of the Coat gene (2226 base pair/742 aa). excluding the stop codon.

C- Numbers refer to values obtained by comparing the complete coding sequences of the RNA-dependent RNA polymerase (2628 base pair/876 aa). excluding the stop codon.

### 5’UTR sequence comparison

5’UTR sequences (from nucleotide 60 to 450) exhibited 86.2 (YA70 *vs*. 2001) to 100% (VL91 *vs*. YZ58 and LF98 *vs*. 2008) nucleotide identities between them and with the LRV1-1 and LRV1-4 5’UTR sequences. Of interest, it should be noted that YA70, which corresponded to the only sequence obtained from a *L*. *(V*.*) braziliensis* isolate, was the most divergent sequence. If we exclude it from the data set, the nucleotide sequence conservation between LRV1 5’UTR sequences from *L*. *(V*.*) guyanensis* isolates ranged from 88.8 to 100%. The same analysis restricted to the LRV1 strains from *L*. *(V*.*) guyanensis* from French Guiana (excluding strain 2001, for which the geographic location of contamination was reported as the region of Manaus, Amazonas state, Brazil) demonstrated a nucleotide sequence conservation ranging from 91.1% to 100%. In addition, taking into account all the sequences analyzed, 74.7% of the 5’UTR nucleotide positions were identical. By excluding YA70, this percentage increased to 79.1%. Again, by restricting our analysis to LRV1 5’UTR sequences obtained from *L*. *(V*.*) guyanensis* strains from French Guiana, 82.1% of the nucleotide positions were identical. These 100% conserved nucleotide positions were distributed into blocks of different sizes. The longest one, a 50-bp motif, covering nucleotides 313–362, encompassed the previously identified pyrimidine-rich region (333-UUUCUUGUUACUAUU-347), whose first nine nucleotides are complementary to a purine-rich region of the *Leishmania* 18S rRNA [47], as well as the nucleocapsid endoribonuclease cleavage site 314-GAUCˇCGAA-321 [13, 14]. In addition, analysis of the stem-loop IV sequences located upstream, from nucleotide 266 to 281, showed that all but two sequences were 100% conserved and identical to the published one [15]. The only two divergent sequences corresponded to strains 2001 and XJ93_G2_. Strain XJ93_G2_ presented two complementary substitutions at positions U269C and A278G of the stem, while strain 2001 presented one substitution at position A278G able to form a wobble base pair with the uracil at position 269 (all positions are given relative to LRV1-1 sequence, accession number NC_002063). This shows that LRV1 5’UTR sequences are strongly conserved in both nucleotide sequences and predicted RNA secondary structures.

### Phylogenies

**Fig 1:**
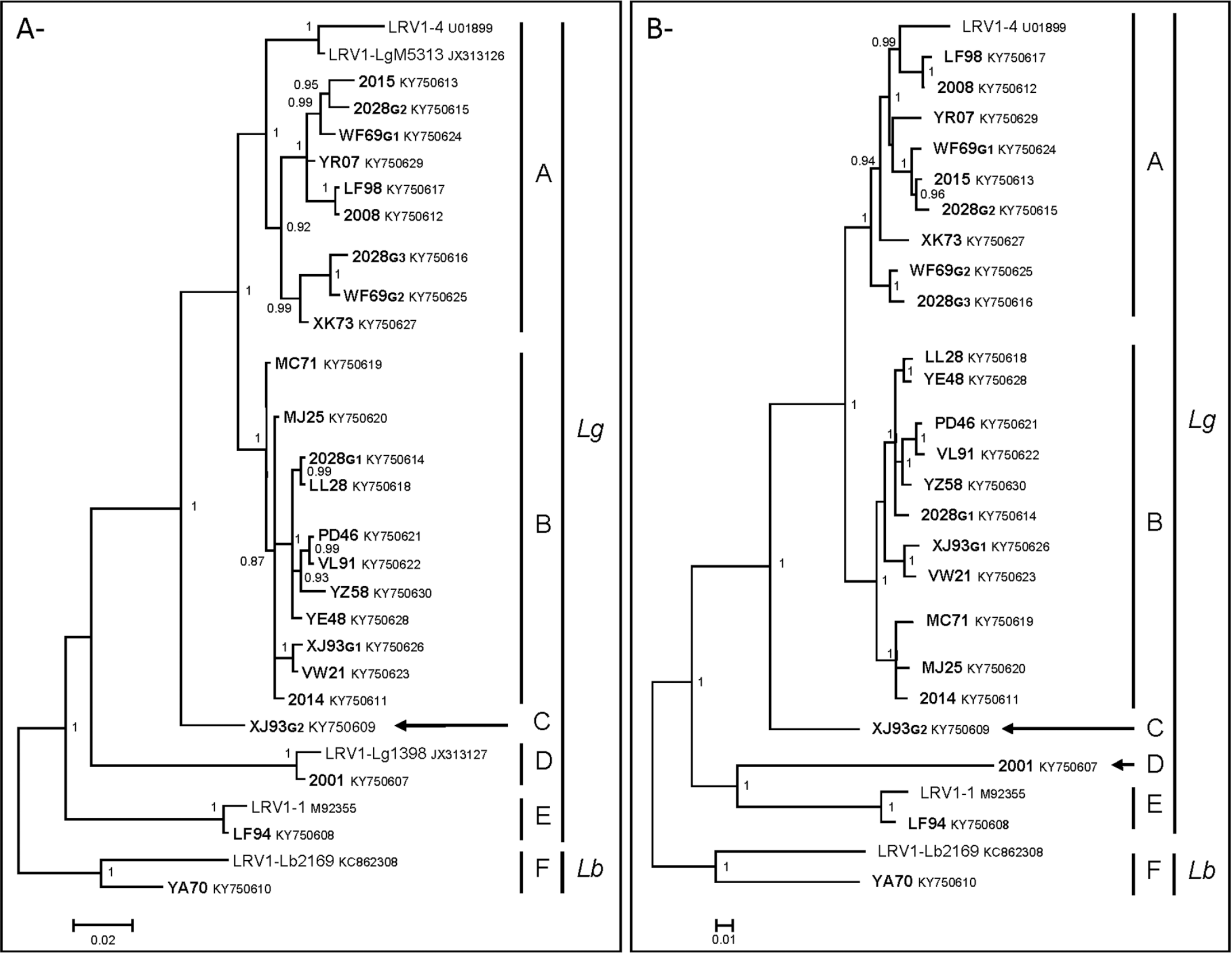
Phylogenetic analysis of *Leishmania* RNA virus 1 isolates. The phylogenetic trees were inferred from A-) the complete amino acid sequences of the *capsid* gene (757 amino acids) and B-) the complete amino acid sequences of the *RdRp* gene (879 amino acids) using the Bayesian method with the JTT + G model of amino acid evolution. New LRV1 sequences generated in this study are in boldface. Sequence identifiers include the strain ID and the NCBI accession number. The major clades representing the different LRV1 genotypes identified (A–F) as well as the clades of LRV1 isolates from *L*. *(V*.*) guyanensis* (*Lg*) or *L*. *(V*.*) braziliensis* (*Lb*) parasites are labeled. Posterior probabilities of the Bayesian analysis (>90%) are shown next to each node. The scale bars indicate amino acid substitutions per site.

Phylogenetic analyses were done on the almost-complete genome sequences (nucleotides 60–5248 relative to the LRV1-1 sequence) (**S1 Fig**), as well as on either nucleotide or amino acid sequences of the capsid (**Fig 1A**) and RNA-dependent RNA polymerase (**Fig 1B**). They all gave strictly identical tree topologies. As mentioned above, we identified six monophyletic “clades”, A–F, all supported by high posterior probabilities. Most of our strains clustered in clades A and B. Indeed, nine of the strains, with LRV1-4 and LRV1-LgM5313, belonged to the monophyletic clade A while clade B was composed of 11 of our strains. All LRV1 strains belonging to clades A and B were from *L*. *(V*.*) guyanensis* parasites. Clades A and B belonged to a monophyletic lineage supported by a posterior probability value of 1. In addition, in clade A, LRV1-LgM5313 and LRV1-4 sequences formed a monophyletic well-supported branch in clade A (**Fig 1A**). These two sequences correspond to Brazilian *L*. *(V*.*) guyanensis* isolates, whereas the others were from French Guiana. Clade C contained only a new divergent strain, XJ93_G2_, which is well supported phylogenetically in all analyses, while clade D was composed of strains 2001 and LRV1-Lg1398. In addition, a phylogenetic analysis restricted to a common 219-bp fragment of the 5’UTR region (**S2 Fig**), which allowed including sequences from most of the LRV1-1 to LRV1-13 strains (except LRV1-3, -5, -6 and -12 for which no sequence is available), demonstrated that strain 2001 was close to LRV1-8 and LRV1-9 strains. These four strains were identified from *L*. *(V*.*) guyanensis* isolates from Brazil. Clade E comprised two strains, one of our sequences, LF94, and LRV1-1. Finally, YA70 and LRV1-Lb2169, identified from *L*. *(V*.*) braziliensis* strains, belonged to a separate clade, F, distinct from the other clades composed of LRV1 sequences of *L*. *(V*.*) guyanensis* (**Fig 1**). Interestingly enough, the two viral sequences identified from WF69 belonged to two distinct groups of sequences within clade A, two of the three strains from 2028 (2028_G2_ and 2028_G3_) were also part of two distinct groups of sequences within clade A, while the third sequence, 2028_G1_, belonged to clade B. Finally, XJ93_G1_ belonged to clade B and XJ93_G2_ was the only representative of clade C.

**Fig 2:**
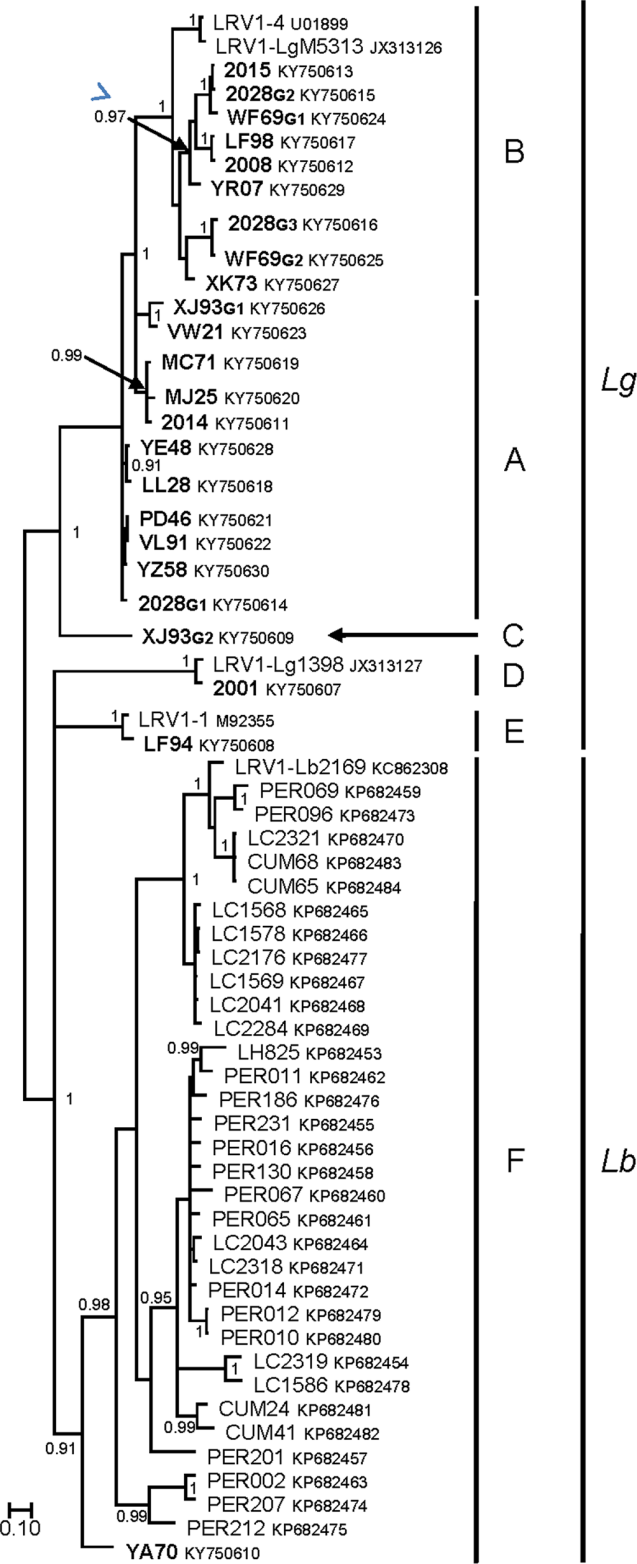
Phylogenetic analysis of partial *capsid* gene sequences (alignment of 299 nucleotides). The tree was inferred using the Bayesian method with the GTR + G + I model. Novel sequences generated in this study are shown in bold. Sequence identifiers include the strain ID and the NCBI accession number. The major clades representing the different LRV1 genotypes identified (A–F) as well as the clades of LRV1 isolates from *L*. *(V*.*) guyanensis* (*Lg*) or *L*. *(V*.*) braziliensis* (*Lb*) parasites are labeled. Posterior probabilities of the Bayesian analysis (>90%) are shown next to each node. The scale bar indicates nucleotide sequence divergence among sequences.

The last phylogenetic analysis, restricted to partial capsid sequences (299bp/99aa), allowed including sequences recently published by Adaui et al. obtained from *L*. *(V*.*) braziliensis* isolates from Peru and Bolivia [29]. All of the LRV1 sequences from *L*. *(V*.*) braziliensis* isolates formed a distinct monophyletic clade, highly supported by a posterior probability of 0.91 (**Fig 2**). LRV1 sequences from *L*. *(V*.*) braziliensis* isolates were subdivided into distinct clades as observed for those from *L*. *(V*.*) guyanensis*. The two sequences previously identified as clade F (YA70 and LRV1-Lb2169) grouped with the other sequences from *L*. *(V*.*) braziliensis* isolates. Interestingly, YA70, which corresponded to the unique strain obtained from a *L*. *(V*.*) braziliensis* isolate from French Guiana, possessed a basal position to the clade. Nevertheless, phylogenetic relationships of LRV1 isolates from *L*. *(V*.*) guyanensis* strains were less robust given that clades D and E clustered with clade F of *L (V*.*) braziliensis* viruses.

### Biological cloning

To determine if the multiple LRV1 strains identified in three *Leishmania* isolates were real viral co-infections or due to the simultaneous presence of distinct parasite populations in our cultures, we proceeded to the biological cloning of the parasites. Two of the three parasites, XJ93 and WF69, were tested. The initial parasite cultures were divided for either RNA isolation or biological cloning. Seven *Leishmania* clones and three subclones of one of the clones were obtained from XJ93. Five clones were obtained from WF69. RNA extracted from the initial cultures and from the different clones and subclones was then submitted to RT-PCR using different combinations of primers, each one specific to a virus (**Table 2**). RT-PCR proved the presence of co-occurring viruses in the initial cultures as well as in all the different clones and subclones.

## Discussion

Herein we present the full-length coding sequences of 24 LRV1 strains obtained from 18 *L*. *(V*.*) guyanensis* and one *L*. *(V*.*) braziliensis* isolates from across French Guiana as well as one *L*. *(V*.*) guyanensis* isolate from the Manaus area, Amazonas state, Brazil. This sequence data set is the largest analysis of LRV1 sequences undertaken so far. Analysis of these sequence data reveals that they have the same genomic organization. With the exception of a few indels detected in five strains, the 5’UTR sequences show, as previously reported, a higher degree of nucleotide sequence conservation than the coding sequences [47]. This high level of sequence conservation, with long– 100% conserved–motifs (up to 50 bp in length), emphasizes the biological importance of this region in the viral life cycle and for maintaining persistent infection [13, 15, 47]. Moreover, only two sequences possessed indels in the coding sequence of the RNA-dependent RNA polymerase. Two of the three indels observed were located at the same position between the two strains. Given that both strains derived from the only two *L*. *(V*.*) braziliensis* isolates for which complete cds of the RdRp are available, these indels could correspond to a molecular signature of LRV1 strains from *L*. *(V*.*) braziliensis*. Further studies are necessary to confirm this point.

From a phylogenetic perspective, it appears that differential clustering exists between viral genomes of *L*. *(V*.*) guyanensis* and *L*. *(V*.*) braziliensis* isolates and among viral genomes of *L*. *(V*.*) guyanensis* isolates. These results support the hypothesis that LRV1 is an ancient virus that has co-evolved with its parasite [25]. In addition, the intra- and inter-clade variability is, overall, below and above 10%, respectively, at both the nucleotide and amino acid levels. Finally, geographical clustering is observed with some clades related to the supposed geographical origin of the parasites. Nevertheless, insufficient geographical sampling, with many areas/countries lacking sufficient data, only based on very short sequences, currently limits phylogeographic interpretations. These results emphasize the crucial need for a better assessment of LRV1 distribution and genetic variation in the different parasite species at a wider geographical scale. Analysis of other *L*. *(V*.*) guyanensis* isolates from different geographical regions as well as of other New World *Leishmania* parasite species should thus expand our understanding of the diversification processes and the evolutionary history of LRV1. These results are also of major importance from a taxonomic viewpoint. They should help define analytical methods and criteria to be used for virus classification and to set up an informative naming system.

A unique viral genome sequence was recovered from 17 of the 20 *Leishmania* isolates tested. Unexpectedly, the three other isolates studied contained more than one viral sequence, indicating that they were co- or super-infected with two or more viruses. The different strains identified from the same isolate showed from about 8% to almost 17% nucleotide divergence among them on the almost complete genome sequences generated. A closer look at the 5’UTR motifs identified as minimal essential elements for site-specific targeting of the capsid endoribonuclease, *i*.*e*. the stem-loop IV structure and single-site specific cleavage site, of the different strains showed their perfect conservation, confirming that they had exact endoribonuclease sites [15]. Furthermore, biological cloning of these parasite isolates followed by RT-PCR using primers specific to each virus confirmed the presence of multiple viruses in each clone. Taken together, these data sustain that the multiple viruses identified in these parasite isolates correspond to true persistent viral co-infections. These results suggest that co- or super-infections with divergent LRV1 strains is common given that three of 20 (15%) isolates were multiply infected. The frequency of co-infections will nevertheless have to be confirmed on a larger series. Furthermore, the relevance of viral co-infection for parasite pathogenesis is unknown. Additional surveys on their biological significance will have to be implemented.

These results provide, moreover, a unique opportunity for implementing reliable diagnostic testing methods. Indeed, knowledge of the genomic sequence of many LRV1 strains was essential for oligonucleotide design ensuring adequate detection of the virus (100% detection rate for LRV1s-LRV2as and 5LRVs1-LRV11as/LRV14as). It is noteworthy that the primers and probes that have previously been described, whether used for quantification or identification of LRV1 in clinical samples, possessed mismatches relative to our sequence data set [22, 26, 29, 32, 48–50]. This could have led to underestimation of the viral load or under-detection of divergent unknown strains. Furthermore, phylogenetic analyses conducted on sequences obtained with our primer pairs (**S3 Fig and S4 Fig**) gave an identical tree topology to those based on complete coding sequences of CP or RdRp. These couples of consensus-degenerate primers are thus robust diagnostic tools intended to be used for future screening of large panels of clinical isolates as well as for molecular phylogenetic studies. Finally, characterization of highly conserved sequence motifs, especially in the 5’UTR region, should help, by defining primers and probes with no mismatch relative to the available sequence data set, implement a pan-LRV1 real-time quantitative reverse-transcription (qRT)-PCR assay for unbiased quantitation of viral RNA. Therefore, given previous results by Ives et al. showing in murine models that a high LRV1 burden, eliciting a high pro-inflammatory profile, was associated with metastasizing parasites, it will be of particular interest to analyze various types of clinical samples to further define the role of LRV1 viral load in parasite virulence, disease progression and response to treatment [28]. These tools should contribute to drawing a more definite causal relationship of LRV1 in disease manifestation.

## Supporting information

S1 Fig Phylogenetic analysis of *Leishmania* RNA virus 1 isolates based on the almost complete genome sequences. The phylogenetic trees were inferred from the almost complete nucleotide sequences (nucleotides 60–5248 relative to the LRV1-1 sequence) using the Bayesian method with the GTR + G + I model. New LRV1 sequences generated in this study are in boldface. Sequence identifiers include the strain ID and the NCBI accession number. The major clades representing the different LRV1 genotypes identified (A–F) are labeled. Posterior probabilities of the Bayesian analysis (>80%) are shown next to each node. The scale bar indicates nucleotide sequence divergence among sequences.
(PDF)

S2 Fig Phylogenetic analysis of partial 5’UTR sequences (alignment of 219 nucleotides). The tree was inferred using the Bayesian method with the GTR + G model. Novel sequences generated in this study are shown in bold. Sequence identifiers include the strain ID and the NCBI accession number. The major clades representing the different LRV1 genotypes identified (A–F) are labeled. Posterior probabilities of the Bayesian analysis (>80%) are shown next to each node. The scale bar indicates nucleotide sequence divergence among sequences.
(PDF)

S3 Fig Phylogenetic analysis of partial 5’UTR—*capsid* gene sequences (alignment of 719 nucleotides corresponding to LRV1s/LRV2as PCR products excluding primers). The tree was inferred using the Bayesian method with the GTR + G + I model. New LRV1 sequences generated in this study are in boldface. Sequence identifiers include the strain ID and the NCBI accession number. The major clades representing the different LRV1 genotypes identified (A–F) are labeled. Posterior probabilities of the Bayesian analysis (>80%) are shown next to each node. The scale bar indicates nucleotide sequence divergence among sequences.
(PDF)

S4 Fig Phylogenetic analysis of partial *RdRp* gene sequences (alignment of 865 nucleotides corresponding to LRV3s/LRV4as PCR products excluding primers). The tree was inferred using the Bayesian method with the T92 +G + I model. New LRV1 sequences generated in this study are in boldface. Sequence identifiers include the strain ID and the NCBI accession number. The major clades representing the different LRV1 genotypes identified (A–F) are labeled. Posterior probabilities of the Bayesian analysis (>80%) are shown next to each node. The scale bar indicates nucleotide sequence divergence among sequences.
(PDF)

## References

[1] Alvar J, Velez ID, Bern C, Herrero M, Desjeux P, Cano J, et al Leishmaniasis worldwide and global estimates of its incidence. PloS one. 2012;7(5):e35671 Pubmed Central PMCID: 3365071. doi: 10.1371/journal.pone.0035671 . 22693548 10.1371/journal.pone.0035671 PMC3365071

[2] David CV, Craft N. Cutaneous and mucocutaneous leishmaniasis. Dermatologic therapy. 2009 Nov-Dec;22(6):491–502. doi: 10.1111/j.1529-8019.2009.01272.x . 19889134 10.1111/j.1529-8019.2009.01272.x

[3] Tarr PI, Aline RF Jr., Smiley BL, Scholler J, Keithly J, Stuart K. LR1: a candidate RNA virus of Leishmania. Proceedings of the National Academy of Sciences of the United States of America. 1988 12;85(24):9572–5. . Pubmed Central PMCID: 282800. 3200841 10.1073/pnas.85.24.9572 PMC282800

[4] Wang AL, Wang CC. Discovery of a specific double-stranded RNA virus in Giardia lamblia. Molecular and biochemical parasitology. 1986 12;21(3):269–76. . 3807947 10.1016/0166-6851(86)90132-5

[5] Wang AL, Wang CC. The double-stranded RNA in Trichomonas vaginalis may originate from virus-like particles. Proceedings of the National Academy of Sciences of the United States of America. 1986 10;83(20):7956–60. . Pubmed Central PMCID: 386843. 3489942 10.1073/pnas.83.20.7956 PMC386843

[6] Altschul SF, Gish W, Miller W, Myers EW, Lipman DJ. Basic local alignment search tool. Journal of molecular biology. 1990 10 5;215(3):403–10. doi: 10.1016/S0022-2836(05)80360-2 . 2231712 10.1016/S0022-2836(05)80360-2

[7] Revets H, Dekegel D, Deleersnijder W, De Jonckheere J, Peeters J, Leysen E, et al Identification of virus-like particles in Eimeria stiedae. Molecular and biochemical parasitology. 1989 10;36(3):209–15. . 2797059 10.1016/0166-6851(89)90168-0

[8] Widmer G, Comeau AM, Furlong DB, Wirth DF, Patterson JL. Characterization of a RNA virus from the parasite Leishmania. Proceedings of the National Academy of Sciences of the United States of America. 1989 8;86(15):5979–82. . Pubmed Central PMCID: 297755. 2762308 10.1073/pnas.86.15.5979 PMC297755

[9] Wang AL, Wang CC. Viruses of parasitic protozoa. Parasitology today. 1991 4;7(4):76–80. . 15463448 10.1016/0169-4758(91)90198-w

[10] Stuart KD, Weeks R, Guilbride L, Myler PJ. Molecular organization of Leishmania RNA virus 1. Proceedings of the National Academy of Sciences of the United States of America. 1992 9 15;89(18):8596–600. . Pubmed Central PMCID: 49967. 1382295 10.1073/pnas.89.18.8596 PMC49967

[11] Maga JA, Widmer G, LeBowitz JH. Leishmania RNA virus 1-mediated cap-independent translation. Molecular and cellular biology. 1995 9;15(9):4884–9. . Pubmed Central PMCID: 230734. 7651407 10.1128/mcb.15.9.4884 PMC230734

[12] Scheffter S, Widmer G, Patterson JL. Complete sequence of Leishmania RNA virus 1–4 and identification of conserved sequences. Virology. 1994 3;199(2):479–83. doi: 10.1006/viro.1994.1149 . 8122377 10.1006/viro.1994.1149

[13] MacBeth KJ, Patterson JL. Single-site cleavage in the 5'-untranslated region of Leishmaniavirus RNA is mediated by the viral capsid protein. Proceedings of the National Academy of Sciences of the United States of America. 1995 9 12;92(19):8994–8. . Pubmed Central PMCID: 41094. 7568059 10.1073/pnas.92.19.8994 PMC41094

[14] MacBeth KJ, Patterson JL. The short transcript of Leishmania RNA virus is generated by RNA cleavage. Journal of virology. 1995 6;69(6):3458–64. . Pubmed Central PMCID: 189058. 7745692 10.1128/jvi.69.6.3458-3464.1995 PMC189058

[15] Ro YT, Patterson JL. Identification of the minimal essential RNA sequences responsible for site-specific targeting of the Leishmania RNA virus 1–4 capsid endoribonuclease. Journal of virology. 2000 1;74(1):130–8. . Pubmed Central PMCID: 111521. 10590099 10.1128/jvi.74.1.130-138.2000 PMC111521

[16] Molyneux DH. Virus-like particles in Leishmania parasites. Nature. 1974 6 7;249(457):588–9. . 4834085 10.1038/249588a0

[17] Cadd TL, Keenan MC, Patterson JL. Detection of Leishmania RNA virus 1 proteins. Journal of virology. 1993 9;67(9):5647–50. . Pubmed Central PMCID: 237969. 8350417 10.1128/jvi.67.9.5647-5650.1993 PMC237969

[18] Salinas G, Zamora M, Stuart K, Saravia N. Leishmania RNA viruses in Leishmania of the Viannia subgenus. The American journal of tropical medicine and hygiene. 1996 4;54(4):425–9. . 8615459 10.4269/ajtmh.1996.54.425

[19] Saiz M, Llanos-Cuentas A, Echevarria J, Roncal N, Cruz M, Muniz MT, et al Short report: detection of Leishmaniavirus in human biopsy samples of leishmaniasis from Peru. The American journal of tropical medicine and hygiene. 1998 2;58(2):192–4. . 9502603 10.4269/ajtmh.1998.58.192

[20] Guilbride L, Myler PJ, Stuart K. Distribution and sequence divergence of LRV1 viruses among different Leishmania species. Molecular and biochemical parasitology. 1992 8;54(1):101–4. . 1518522 10.1016/0166-6851(92)90099-6

[21] Ginouves M, Simon S, Bourreau E, Lacoste V, Ronet C, Couppie P, et al Prevalence and Distribution of Leishmania RNA Virus 1 in Leishmania Parasites from French Guiana. The American journal of tropical medicine and hygiene. 2016 1 6;94(1):102–6. doi: 10.4269/ajtmh.15-0419 . 26598572 10.4269/ajtmh.15-0419 PMC4710412

[22] Ogg MM, Carrion R Jr., Botelho AC, Mayrink W, Correa-Oliveira R, Patterson JL. Short report: quantification of leishmaniavirus RNA in clinical samples and its possible role in pathogenesis. The American journal of tropical medicine and hygiene. 2003 9;69(3):309–13. . 14628949

[23] Zangger H, Hailu A, Desponds C, Lye LF, Akopyants NS, Dobson DE, et al Leishmania aethiopica field isolates bearing an endosymbiontic dsRNA virus induce pro-inflammatory cytokine response. PLoS neglected tropical diseases. 2014 4;8(4):e2836 Pubmed Central PMCID: 3998932. doi: 10.1371/journal.pntd.0002836 . 24762979 10.1371/journal.pntd.0002836 PMC3998932

[24] Hajjaran H, Mahdi M, Mohebali M, Samimi-Rad K, Ataei-Pirkooh A, Kazemi-Rad E, et al Detection and molecular identification of leishmania RNA virus (LRV) in Iranian Leishmania species. Archives of virology. 2016 9 7 doi: 10.1007/s00705-016-3044-z . 27604119 10.1007/s00705-016-3044-z

[25] Widmer G, Dooley S. Phylogenetic analysis of Leishmania RNA virus and Leishmania suggests ancient virus-parasite association. Nucleic acids research. 1995 6 25;23(12):2300–4. . Pubmed Central PMCID: 307021. 7610059 10.1093/nar/23.12.2300 PMC307021

[26] Cantanhede LM, da Silva Junior CF, Ito MM, Felipin KP, Nicolete R, Salcedo JM, et al Further Evidence of an Association between the Presence of Leishmania RNA Virus 1 and the Mucosal Manifestations in Tegumentary Leishmaniasis Patients. PLoS neglected tropical diseases. 2015 9;9(9):e0004079 Pubmed Central PMCID: 4570810. doi: 10.1371/journal.pntd.0004079 . 26372217 10.1371/journal.pntd.0004079 PMC4570810

[27] Scheffter SM, Ro YT, Chung IK, Patterson JL. The complete sequence of Leishmania RNA virus LRV2-1, a virus of an Old World parasite strain. Virology. 1995 9 10;212(1):84–90. doi: 10.1006/viro.1995.1456 . 7676652 10.1006/viro.1995.1456

[28] Ives A, Ronet C, Prevel F, Ruzzante G, Fuertes-Marraco S, Schutz F, et al Leishmania RNA virus controls the severity of mucocutaneous leishmaniasis. Science. 2011 2 11;331(6018):775–8. Pubmed Central PMCID: 3253482. doi: 10.1126/science.1199326 . 21311023 10.1126/science.1199326 PMC3253482

[29] Adaui V, Lye LF, Akopyants NS, Zimic M, Llanos-Cuentas A, Garcia L, et al Association of the Endobiont Double-Stranded RNA Virus LRV1 With Treatment Failure for Human Leishmaniasis Caused by Leishmania braziliensis in Peru and Bolivia. The Journal of infectious diseases. 2016 1 1;213(1):112–21. Pubmed Central PMCID: 4676543. doi: 10.1093/infdis/jiv354 . 26123565 10.1093/infdis/jiv354 PMC4676543

[30] Bourreau E, Ginouves M, Prevot G, Hartley MA, Gangneux JP, Robert-Gangneux F, et al Presence of Leishmania RNA Virus 1 in Leishmania guyanensis Increases the Risk of First-Line Treatment Failure and Symptomatic Relapse. The Journal of infectious diseases. 2016 1 1;213(1):105–11. doi: 10.1093/infdis/jiv355 . 26123564 10.1093/infdis/jiv355

[31] Pereira Lde O, Maretti-Mira AC, Rodrigues KM, Lima RB, Oliveira-Neto MP, Cupolillo E, et al Severity of tegumentary leishmaniasis is not exclusively associated with Leishmania RNA virus 1 infection in Brazil. Memorias do Instituto Oswaldo Cruz. 2013 8;108(5):665–7. Pubmed Central PMCID: 3970596. doi: 10.1590/0074-0276108052013021 . 23903986 10.1590/0074-0276108052013021 PMC3970596

[32] Ito MM, Catanhede LM, Katsuragawa TH, da Silva Junior CF, Camargo LM, Mattos Rde G, et al Correlation between presence of Leishmania RNA virus 1 and clinical characteristics of nasal mucosal leishmaniosis. Brazilian journal of otorhinolaryngology. 2015 Sep-Oct;81(5):533–40. doi: 10.1016/j.bjorl.2015.07.014 . 26277588 10.1016/j.bjorl.2015.07.014 PMC9449032

[33] Simon S, Nacher M, Carme B, Basurko C, Roger A, Adenis A, et al Cutaneous leishmaniasis in French Guiana: revising epidemiology with PCR-RFLP. Trop Med Health. 2017;45:5 Epub 2017 Feb 28. doi: 10.1186/s41182-017-0045-x 28265182 10.1186/s41182-017-0045-x PMC5331739

[34] Rotureau B, Ravel C, Nacher M, Couppie P, Curtet I, Dedet JP, et al Molecular epidemiology of Leishmania (Viannia) guyanensis in French Guiana. Journal of clinical microbiology. 2006 2;44(2):468–73. Pubmed Central PMCID: 1392701. doi: 10.1128/JCM.44.2.468-473.2006 . 16455900 10.1128/JCM.44.2.468-473.2006 PMC1392701

[35] Rotureau B, Ravel C, Couppie P, Pratlong F, Nacher M, Dedet JP, et al Use of PCR-restriction fragment length polymorphism analysis to identify the main new world Leishmania species and analyze their taxonomic properties and polymorphism by application of the assay to clinical samples. Journal of clinical microbiology. 2006 2;44(2):459–67. Pubmed Central PMCID: 1392689. doi: 10.1128/JCM.44.2.459-467.2006 . 16455899 10.1128/JCM.44.2.459-467.2006 PMC1392689

[36] Simon S, Veron V, Carme B. Leishmania spp. identification by polymerase chain reaction-restriction fragment length polymorphism analysis and its applications in French Guiana. Diagnostic microbiology and infectious disease. 2010 2;66(2):175–80. doi: 10.1016/j.diagmicrobio.2009.08.013 . 19782495 10.1016/j.diagmicrobio.2009.08.013

[37] Aronesty E. ea-utils: "Command-line tools for processing biological sequencing data" 2011. Available from: http://code.google.com/p/ea-utils.

[38] Schmieder R, Edwards R. Fast identification and removal of sequence contamination from genomic and metagenomic datasets. PloS one. 2011;6(3):e17288 Pubmed Central PMCID: 3052304. doi: 10.1371/journal.pone.0017288 . 21408061 10.1371/journal.pone.0017288 PMC3052304

[39] Bankevich A, Nurk S, Antipov D, Gurevich AA, Dvorkin M, Kulikov AS, et al SPAdes: a new genome assembly algorithm and its applications to single-cell sequencing. Journal of computational biology: a journal of computational molecular cell biology. 2012 5;19(5):455–77. Pubmed Central PMCID: 3342519. doi: 10.1089/cmb.2012.0021 . 22506599 10.1089/cmb.2012.0021 PMC3342519

[40] Li H. Aligning sequence reads, clone sequences and assembly contigs with BWA-MEM. arXiv:1303.3997v2 [q-bio.GN] ed2013.

[41] Katoh K, Standley DM. MAFFT multiple sequence alignment software version 7: improvements in performance and usability. Molecular biology and evolution. 2013 4;30(4):772–80. Pubmed Central PMCID: 3603318. doi: 10.1093/molbev/mst010 . 23329690 10.1093/molbev/mst010 PMC3603318

[42] Tamura K, Peterson D, Peterson N, Stecher G, Nei M, Kumar S. MEGA5: molecular evolutionary genetics analysis using maximum likelihood, evolutionary distance, and maximum parsimony methods. Molecular biology and evolution. 2011 10;28(10):2731–9. Pubmed Central PMCID: 3203626. doi: 10.1093/molbev/msr121 . 21546353 10.1093/molbev/msr121 PMC3203626

[43] Nylander JAA. MrModeltest v2. 2004.

[44] Ronquist F, Teslenko M, van der Mark P, Ayres DL, Darling A, Hohna S, et al MrBayes 3.2: efficient Bayesian phylogenetic inference and model choice across a large model space. Systematic biology. 2012 5;61(3):539–42. Pubmed Central PMCID: 3329765. doi: 10.1093/sysbio/sys029 . 22357727 10.1093/sysbio/sys029 PMC3329765

[45] Darriba D, Taboada GL, Doallo R, Posada D. ProtTest 3: fast selection of best-fit models of protein evolution. Bioinformatics. 2011 4 15;27(8):1164–5. doi: 10.1093/bioinformatics/btr088 . 21335321 10.1093/bioinformatics/btr088 PMC5215816

[46] Adams MJ, Lefkowitz EJ, King AM, Carstens EB. Ratification vote on taxonomic proposals to the International Committee on Taxonomy of Viruses (2014). Archives of virology. 2014 10;159(10):2831–41. doi: 10.1007/s00705-014-2114-3 . 24906522 10.1007/s00705-014-2114-3

[47] Zamora M, Guilbride L, Sacks L, Stuart K. Phylogenetic analysis of the 5' subterminal region of isolates of Leishmania RNA virus-1. Annals of tropical medicine and parasitology. 2000 3;94(2):123–33. . 10827867 10.1080/00034980057464

[48] Macedo DH, Menezes-Neto A, Rugani JM, Rocha AC, Silva SO, Melo MN, et al Low frequency of LRV1 in Leishmania braziliensis strains isolated from typical and atypical lesions in the State of Minas Gerais, Brazil. Molecular and biochemical parasitology. 2016 8 18 doi: 10.1016/j.molbiopara.2016.08.005 . 27546549 10.1016/j.molbiopara.2016.08.005 PMC5125831

[49] Lye LF, Owens K, Shi H, Murta SM, Vieira AC, Turco SJ, et al Retention and loss of RNA interference pathways in trypanosomatid protozoans. PLoS pathogens. 2010;6(10):e1001161 Pubmed Central PMCID: 2965760. doi: 10.1371/journal.ppat.1001161 . 21060810 10.1371/journal.ppat.1001161 PMC2965760

[50] Zangger H, Ronet C, Desponds C, Kuhlmann FM, Robinson J, Hartley MA, et al Detection of Leishmania RNA virus in Leishmania parasites. PLoS neglected tropical diseases. 2013;7(1):e2006 Pubmed Central PMCID: 3542153. doi: 10.1371/journal.pntd.0002006 . 23326619 10.1371/journal.pntd.0002006 PMC3542153

