# Supplementary figures and images for: Unraveling the genetic diversity and phylogeny of Leishmania RNA virus 1 strains of infected Leishmania isolates circulating in French Guiana
Source: PLoS Negl Trop Dis. 2017 Jul 17;11(7):e0005764. doi: 10.1371/journal.pntd.0005764 (PMC5531682; doi:10.1371/journal.pntd.0005764)

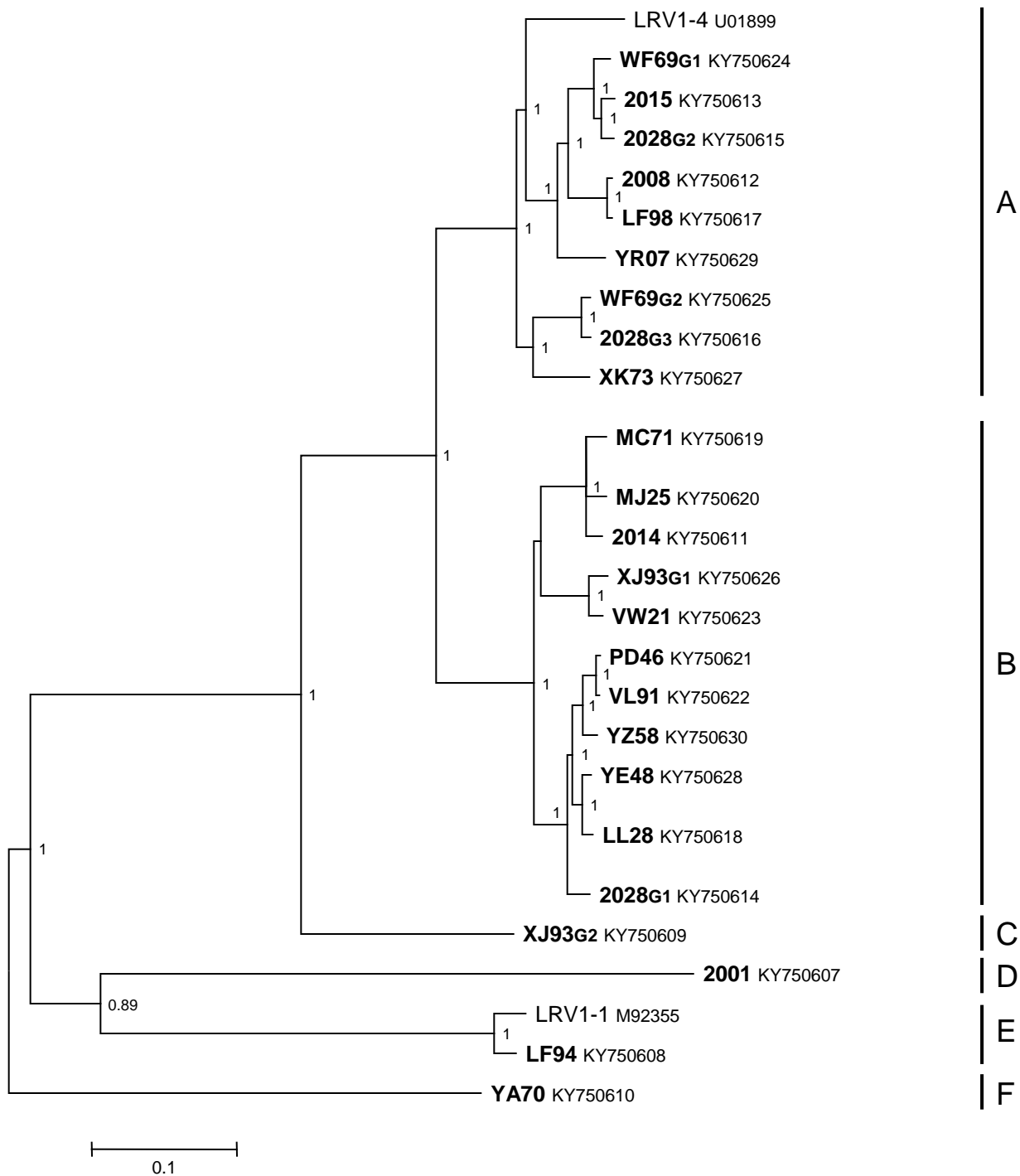

Supplement: S1 Fig — The phylogenetic trees were inferred from the almost complete nucleotide sequences (nucleotides 60–5248 relative to the LRV1-1 sequence) using the Bayesian method with the GTR + G + I model. New LRV1 sequences generated in this study are in boldface. Sequence identifiers include the strain ID and the NCBI accession number. The major clades representing the different LRV1 genotypes identified (A–F) are labeled. Posterior probabilities of the Bayesian analysis (>80%) are shown next to each node. The scale bar indicates nucleotide sequence divergence among sequences. (PDF) [file pntd.0005764.s001.pdf]

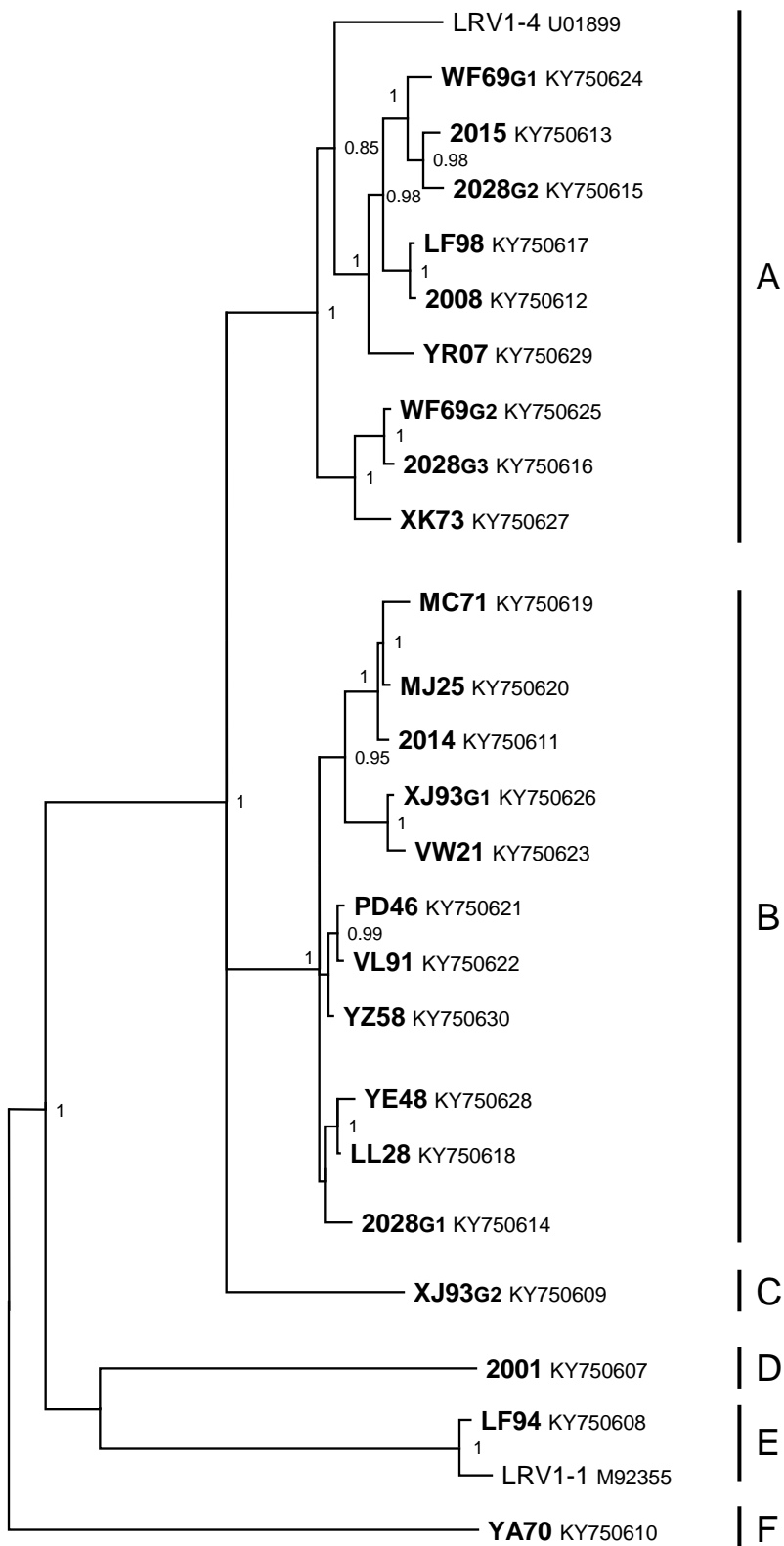

Supplement: S3 Fig — The tree was inferred using the Bayesian method with the GTR + G + I model. New LRV1 sequences generated in this study are in boldface. Sequence identifiers include the strain ID and the NCBI accession number. The major clades representing the different LRV1 genotypes identified (A–F) are labeled. Posterior probabilities of the Bayesian analysis (>80%) are shown next to each node. The scale bar indicates nucleotide sequence divergence among sequences. (PDF) [file pntd.0005764.s003.pdf]

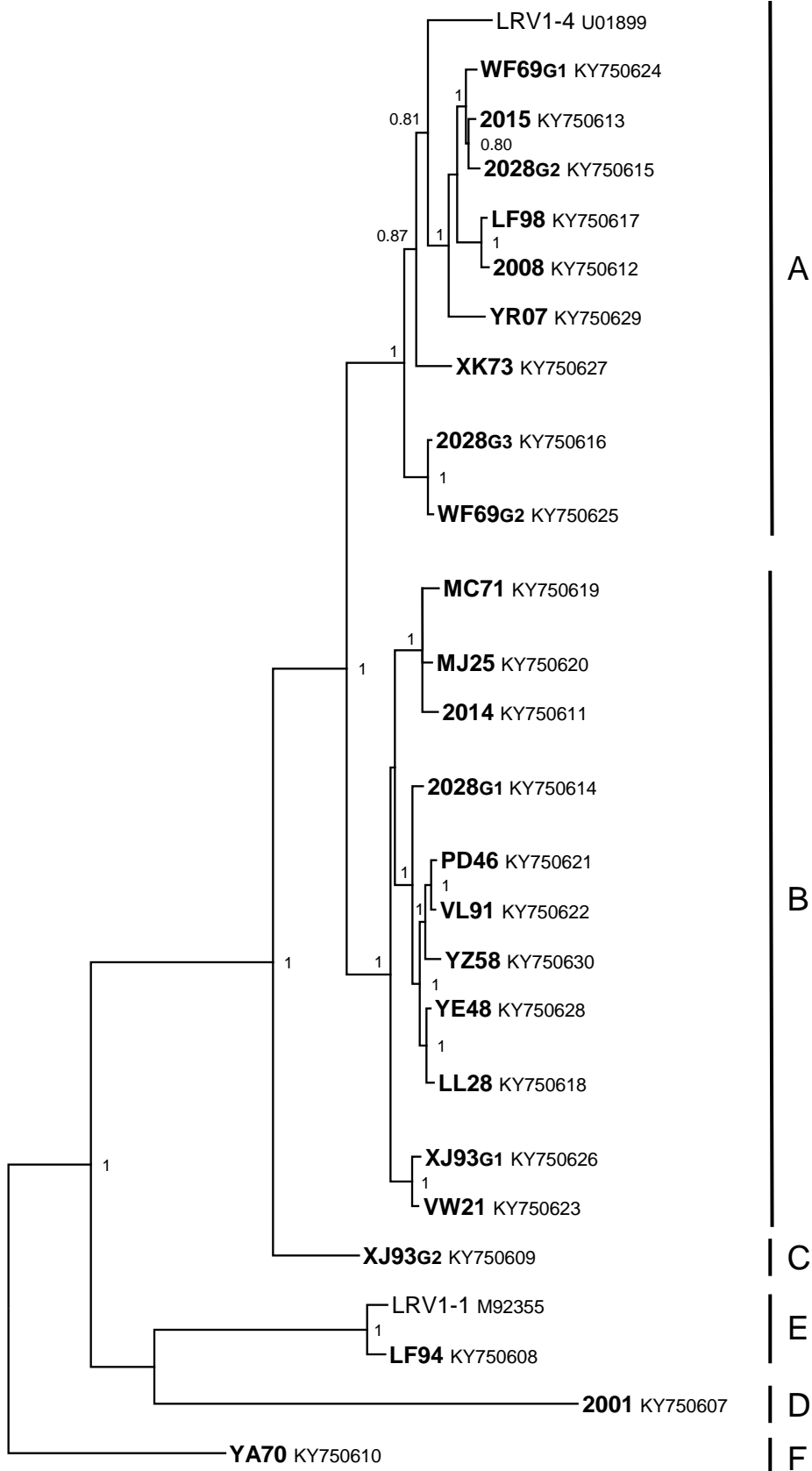

Supplement: S4 Fig — The tree was inferred using the Bayesian method with the T92 +G + I model. New LRV1 sequences generated in this study are in boldface. Sequence identifiers include the strain ID and the NCBI accession number. The major clades representing the different LRV1 genotypes identified (A–F) are labeled. Posterior probabilities of the Bayesian analysis (>80%) are shown next to each node. The scale bar indicates nucleotide sequence divergence among sequences. (PDF) [file pntd.0005764.s004.pdf]
