# Supplementary material for: Unraveling the genetic diversity and phylogeny of Leishmania RNA virus 1 strains of infected Leishmania isolates circulating in French Guiana
Source: PLoS Negl Trop Dis. 2017 Jul 17;11(7):e0005764. doi: 10.1371/journal.pntd.0005764 (PMC5531682; doi:10.1371/journal.pntd.0005764)
Supplement: S2 Fig — The tree was inferred using the Bayesian method with the GTR + G model. Novel sequences generated in this study are shown in bold. Sequence identifiers include the strain ID and the NCBI accession number. The major clades representing the different LRV1 genotypes identified (A–F) are labeled. Posterior probabilities of the Bayesian analysis (>80%) are shown next to each node. The scale bar indicates nucleotide sequence divergence among sequences. (PDF) [file pntd.0005764.s002.pdf]

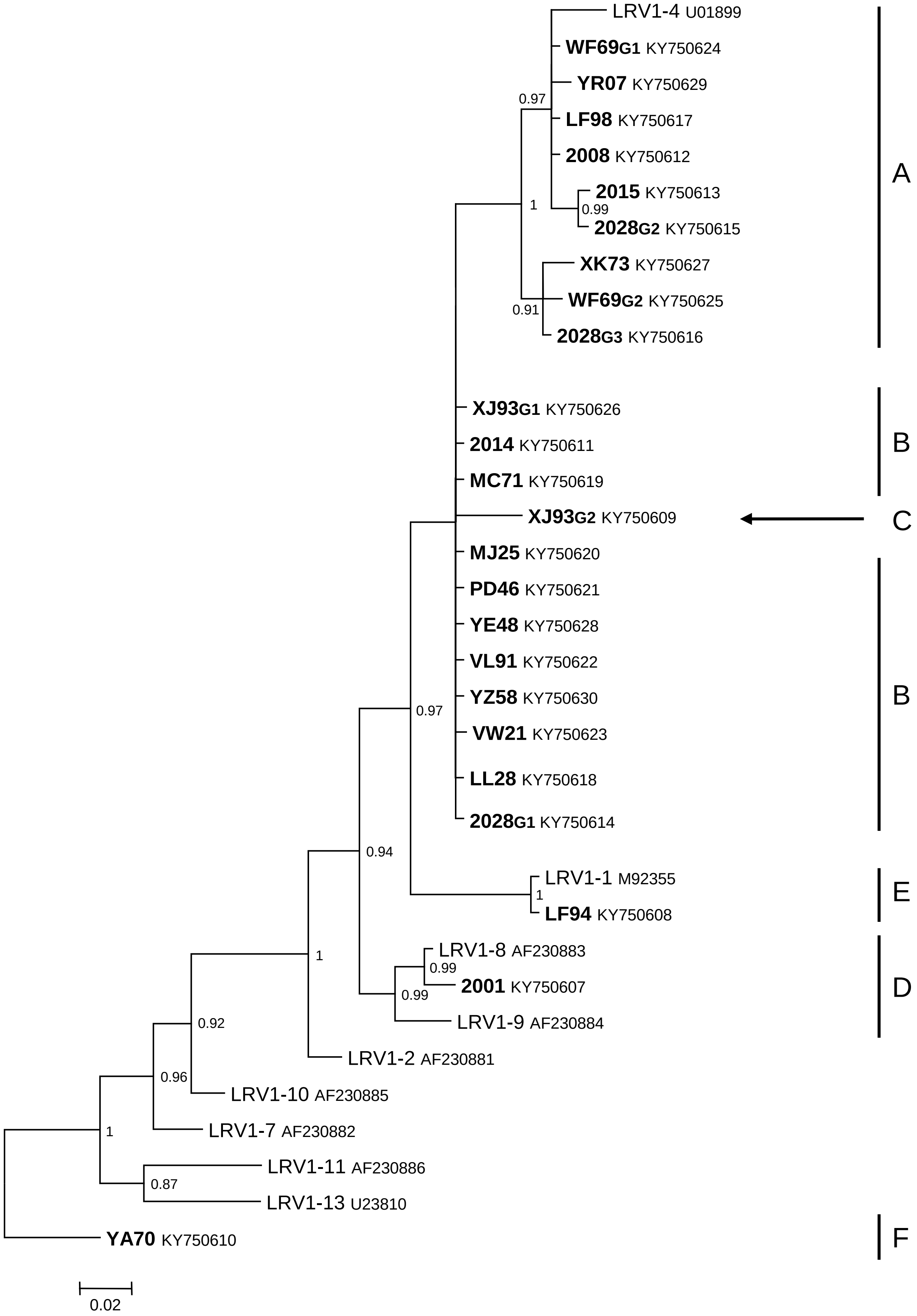
LRV1-4 U01899
WF69G1 KY750624
YR07 KY750629
LF98 KY750617
2008 KY750612
2015 KY750613
2028G2 KY750615
XK73 KY750627
WF69G2 KY750625
2028G3 KY750616
XJ93G1 KY750626
2014 KY750611
MC71 KY750619
XJ93G2 KY750609
MJ25 KY750620
PD46 KY750621
YE48 KY750628
VL91 KY750622
YZ58 KY750630
VW21 KY750623
LL28 KY750618
2028G1 KY750614
LRV1-1 M92355
LF94 KY750608
LRV1-8 AF230883
2001 KY750607
LRV1-9 AF230884
LRV1-2 AF230881
LRV1-10 AF230885
LRV1-7 AF230882
LRV1-11 AF230886
LRV1-13 U23810
YA70 KY750610
0.97
1
0.99
0.91
0.97
0.94
1
0.99
0.99
1
0.92
0.96
1
0.87
A
B
C
B
E
D
F
0.02
